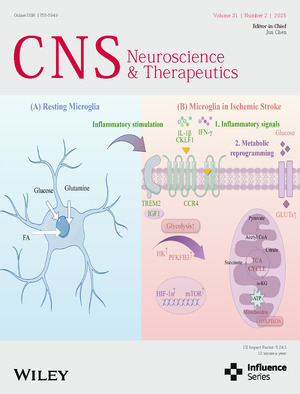# Front Cover

**DOI:** 10.1111/cns.70281

**Published:** 2025-02-26

## Abstract

Cover image: The cover image is based on the article *Examining the Impact of Microglia on Ischemic Stroke With an Emphasis on the Metabolism of Immune Cells* by Xufeng Tao et al., https://doi.org/10.1111/cns.70229.